# Dual function of tropodithietic acid as antibiotic and signaling molecule in global gene regulation of the probiotic bacterium *Phaeobacter inhibens*

**DOI:** 10.1038/s41598-017-00784-7

**Published:** 2017-04-07

**Authors:** Paul G. Beyersmann, Jürgen Tomasch, Kwangmin Son, Roman Stocker, Markus Göker, Irene Wagner-Döbler, Meinhard Simon, Thorsten Brinkhoff

**Affiliations:** 1 Institute for Chemistry and Biology of the Marine Environment (ICBM), University of Oldenburg, 26129 Oldenburg, Germany; 2 Helmholtz Centre for Infection Research, 38124 Braunschweig, Germany; 3 Department of Civil and Environmental Engineering, Massachusetts Institute of Technology, Cambridge, MA 02139 USA; 4 Department of Mechanical Engineering, Massachusetts Institute of Technology, Cambridge, MA 02139 USA; 5 Institute for Environmental Engineering, Department of Civil, Environmental and Geomatic Engineering, Eidgenössische Technische Hochschule (ETH) Zürich, Zurich, 8093 Switzerland; 6 Leibniz-Institute DSMZ – German Collection of Microorganisms and Cell Cultures GmbH, 38124 Braunschweig, Germany

## Abstract

Antibiotics are typically regarded as microbial weapons, but whereas their function at concentrations lethal for bacteria is often well characterized, the role of antibiotics at much lower concentrations as possibly found under natural conditions remains poorly understood. By using whole-transcriptome analyses and phenotypic screenings of the marine bacterium *Phaeobacter inhibens* we found that the broad-spectrum antibiotic tropodithietic acid (TDA) causes the same regulatory effects in quorum sensing (QS) as the common signaling molecule N-acyl-homoserine lactone (AHL) at concentrations 100-fold lower than the minimal inhibitory concentration against bacteria. Our results show that TDA has a significant impact on the expression of ~10% of the total genes of *P. inhibens*, in the same manner as the AHL. Furthermore, TDA needs the AHL associated LuxR-type transcriptional regulator, just as the AHL molecule. Low concentrations of antibiotics can obviously have a strong influence on the global gene expression of the bacterium that produces it and drastically change the metabolism and behaviour of the bacterium. For *P. inhibens* this includes motility, biofilm formation and antibiotic production, all important for settlement on new host-associated surfaces. Our results demonstrate that bacteria can produce antibiotics not only to antagonise other bacteria, but also to mediate QS like endogenous AHL molecules.

## Introduction

Since the discovery of penicillin in 1929, more than 200 antibiotic drugs that are direct natural products have been discovered and their inhibitory bioactivity has been extensively studied^1, 2^. Whereas the function of these antibiotics at concentrations lethal for bacteria is often well characterized, their role in natural environments at sub-inhibitory concentrations remains poorly understood^3^. In recent years it was proposed that antibiotics may not only serve as killing or growth-inhibitory compounds, but also function as inter-microbial signals even below the minimal inhibitory concentration^3–7^. Antibiotics at sub-inhibitory concentrations have been found to interfere with bacterial cell regulation systems, altering global transcription patterns and affecting up to 5–10% of all transcripts^4, 8–10^, including regulators involved in quorum sensing (QS). QS is a cell-to-cell communication system mediated by signaling molecules inducing expression of defined sets of genes, e.g., encoding for energy-intensive processes like biofilm formation, cell motility and antibiotic production, and hypothesized to facilitate adaptations to environmental stimuli^11–13^. The addition of antibiotics to *Chromobacterium violaceum* cultures enhanced production of the QS signaling molecules *N*-acyl-homoserine lactones (AHLs). Because of the diverse molecular structures of antibiotics, however, the possibility that they act as AHL analogues was previously ruled out^6^. Our data demonstrate, instead, that the antibiotic tropodithietic acid (TDA) can act like the endogenous AHL in the marine bacterium *Phaeobacter inhibens* in triggering QS.

*Phaeobacter inhibens* is a strong and competitive biofilm producer^14^, found to be associated with various eukaryotic organisms, and is a member of the *Roseobacter* group (family *Rhodobacteraceae*, *Alphaproteobacteria*), which is highly abundant in marine habitats^15, 16^. *Phaeobacter inhibens* as well as members of the genera *Ruegeria* and *Pseudovibrio* can produce TDA^17^, however, to this day, only a few TDA-tolerant strains have been found in the natural environment, co-occurring with TDA producers^18^. TDA has anticancer activities and the mode of action of TDA is similar to that of large polyether antibiotics, which work by disrupting the proton motive force^19^. Thus, there is an increasing interest in TDA as an antibiotic and in *Phaeobacter* as a probiotic organism in aquacultures and other applications^20–22^.

Production of TDA has been described for various genera, able to increase TDA gene expression by cross-feeding, suggesting that TDA functions as an autoinducer of its own synthesis^23^. Presence of genes coding for diguanylyl cyclases and c-di-GMP (cyclic dimeric guanosinmonophosphate)-specific phosphodiesterases in *P. inhibens* and other roseobacters suggests that c-di-GMP signalling is a universal feature of this group, and TDA production in *Ruegeria mobilis* was found to be influenced by intracellular c-di-GMP concentrations^24^. It was suggested that in the *Roseobacter* clade intra and extracellular cues are integrated via a c-di-GMP second messenger system and that expression of phenotypic traits specific for either planktonic or attached life is regulated in response to c-di-GMP concentrations^24^.

Biosynthesis of TDA in *P. inhibens* is regulated by AHL-mediated QS based on a *luxIR* homologous system with *pgaI* encoding for the N-3-hydroxydecanoylhomoserine lactone (3OHC(10)-HSL) synthase, and *pgaR* for the corresponding AHL response regulator^17^. Mutants of these genes (*pgaI* ^*−*^ and *pgaR* ^*−*^) were deficient in TDA production. TDA at sub-inhibitory concentrations restored TDA gene expression in *pgaI* ^*−*^, but not in *pgaR* ^*−*^, confirming the role of TDA as an autoinducer^17^. This prompted us to consider whether TDA regulates more than its own biosynthesis. Using whole-transcriptome analyses and phenotypic screenings, we show that gene expression and gene functions are mediated by both TDA and AHL, and demonstrate that production of TDA in *P. inhibens* can substitute production of its AHL.

## Results

### Global gene regulation by AHL-mediated QS

**Figure 1:**
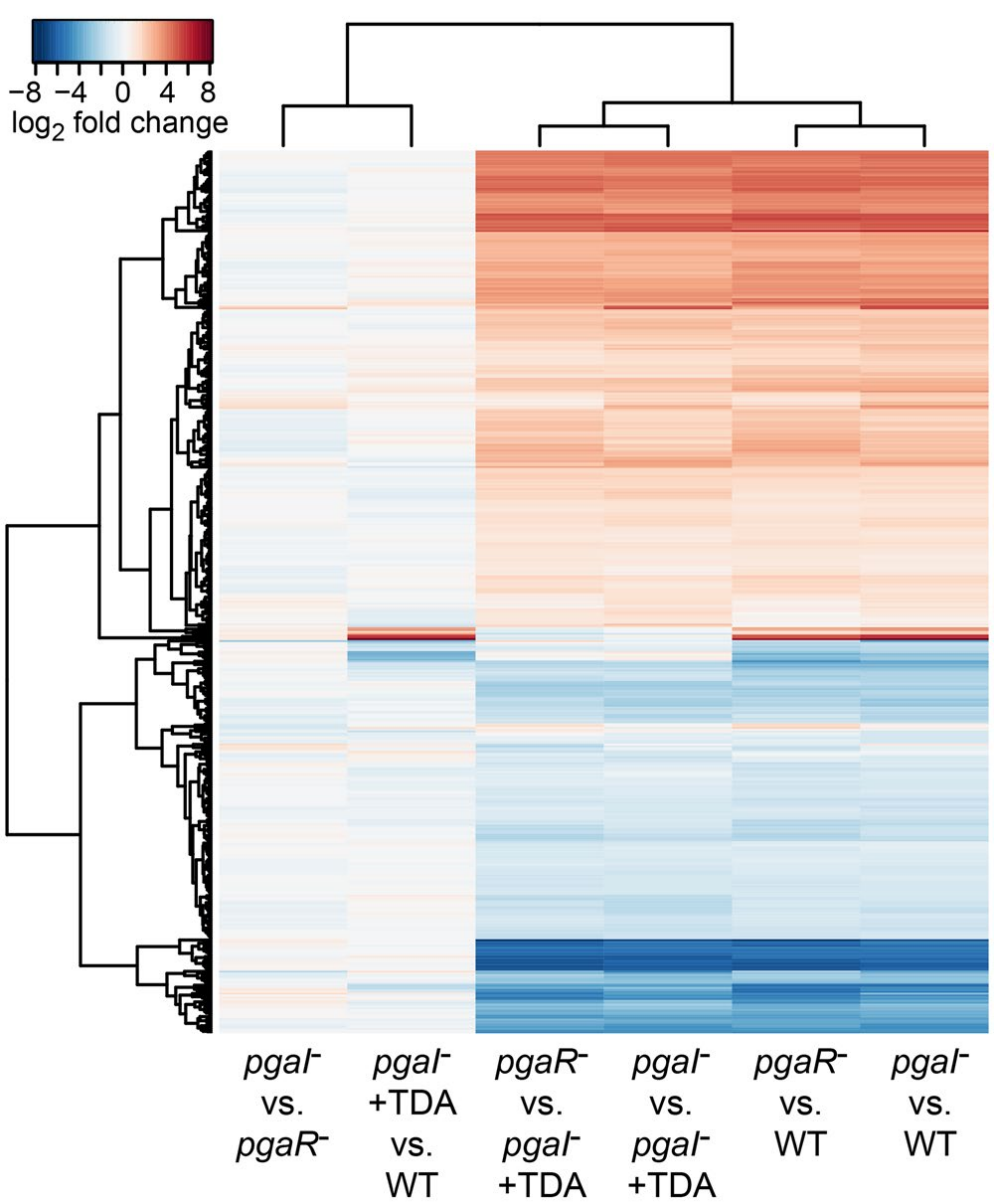
Hierarchical clustering based on gene expression patterns of WT and mutants of *P. inhibens* analysed by whole transcriptome microarrays. In each column two transcriptomes are compared. “+TDA” indicates addition of subinhitory amounts of TDA to the culture (Methods). Identical genes are at the same height in each column and clustered in height based expression fold changes. Red indicates upregulated, blue downregulated gene expression of the upper strain with respect to the lower strain listed below the respective column. Dendrograms are based on euclidean distances and provide a measure of relatedness, at the left side for clustered differentially expressed genes and at the top for the different microarrays. Minor differences between the two microarrays “*pgaI* ^*−*^ vs. WT” and “*pgaR* ^*−*^ vs. WT” are largely based on statistical calculations including cut-off values (absolute value of fold change ≥1, *p*-value < 0.01).

Compared to the transcriptome of the *P. inhibens* wild type strain (WT), lack of the AHL molecule or of the AHL regulator significantly changed gene expression, with 410 genes affected in *pgaI* ^*−*^ and 326 in *pgaR* ^*−*^ (*p* ≤ 0.01), corresponding to 10.5% and 8.4% of the total genes, respectively (Fig. 1, Table S1). The expression patterns for “*pgaI* ^*−*^ vs. WT” and “*pgaR* ^*−*^ vs. WT” showed almost the same result (Fig. 1), with 307 identical genes differentially expressed in *pgaI* ^*−*^ and in *pgaR* ^*−*^ compared to the transcriptome of the WT (Table S1). When compared to each other, the transcriptomes of both mutants showed only one differentially expressed gene, namely *pgaI*, explained by the insertion in *pgaI* ^*−*^. Thus, the corresponding AHL and AHL regulator co-mediate QS in *P. inhibens* and regulate the same ~10% of the genome.

### Global gene regulation by TDA-mediated QS

By adding sub-inhibitory concentrations of TDA to the AHL synthase-deficient mutant of *P. inhibens* (*pgaI* ^*−*^ + TDA), we studied whether TDA influences expression of more genes than those encoding for its own biosynthesis. Typical minimum inhibitory concentrations (MIC) of TDA against diverse bacteria range from 188.5 µM to 5.9 mM^20^. At ca. 1 mM, TDA also starts to inhibit *Phaeobacter* spp^20, 25^. Here we used a TDA concentration of 1.5 µM, >100-fold lower than typical MICs for this antibiotic.

The influence of TDA on global gene regulation is apparent from the comparison of transcriptomes of *pgaI* ^*−*^ and *pgaI* ^*−*^ + TDA, which showed 297 genes differentially expressed (Fig. 1, Table S1). Of these genes, 274 are also differentially expressed in the “*pgaI* ^*−*^ vs. WT” microarray and 241 in the “*pgaR* ^*−*^ vs. WT” microarray. Importantly, a comparison of the whole transcriptomes of *pgaI* ^*−*^ + TDA and the WT revealed that only 15 genes were differentially expressed (Fig. 1, Table S1). The whole transcriptomes of the *pgaI* ^*−*^ + TDA and the WT are highly correlated with each other, but not with the transcriptomes of the two mutants (*pgaI* ^*−*^ and *pgaR* ^*−*^), which are highly correlated with each other, too (Fig. S1). This demonstrates that exogenous TDA largely restored the WT transcriptome in *pgaI* ^*−*^ in the late exponential growth phase. Results derived from comparisons of the transcriptomes of both mutants either with that of the WT or that of the *pgaI* ^*−*^ + TDA highly resemble each other (Fig. 1). This shows that TDA substitutes the lacking AHL in *pgaI* ^*−*^ and thus has an influence on global gene expression in a manner very similar to the AHL molecule.

### Specific gene functions regulated by AHL and TDA

**Figure 2:**
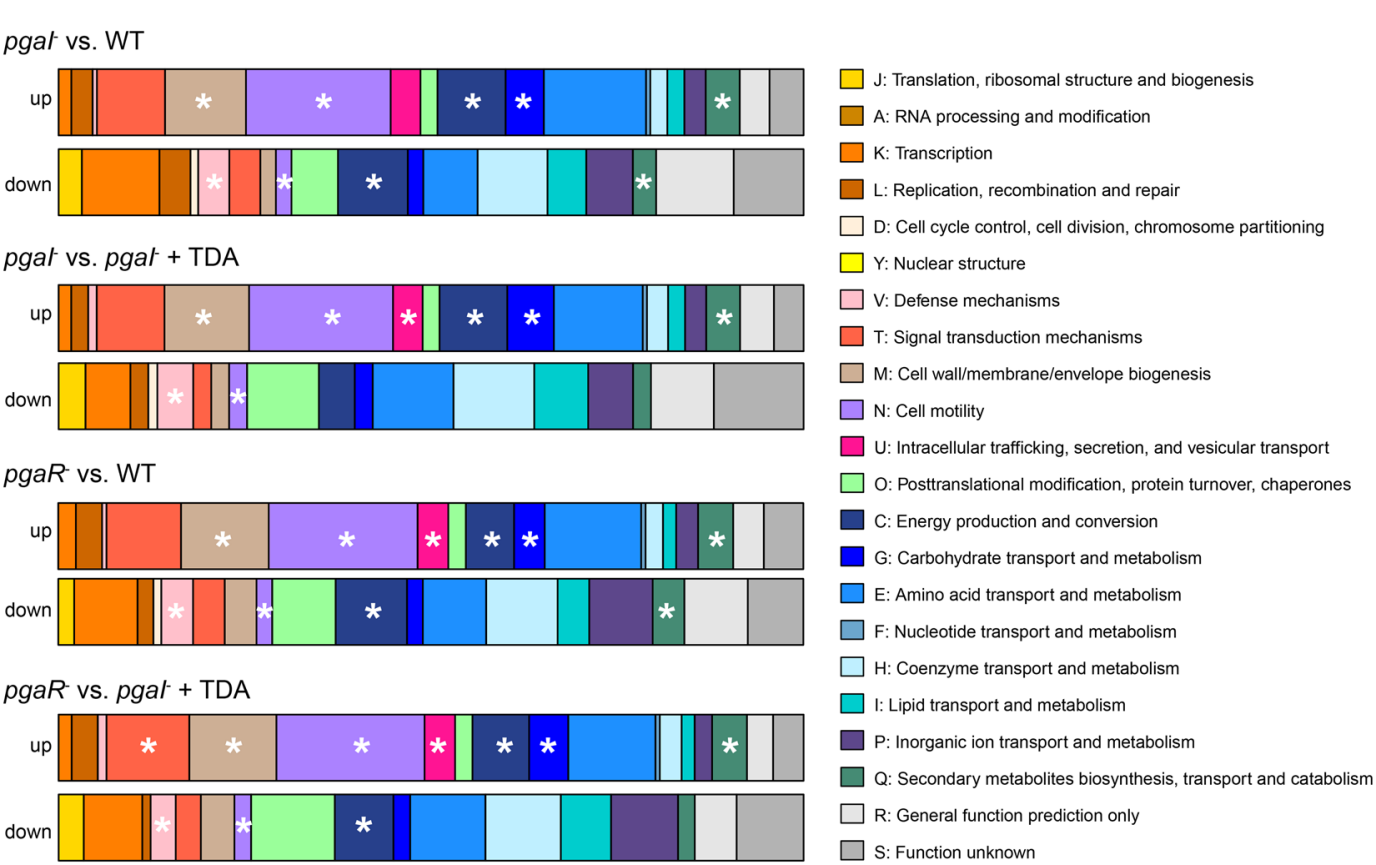
Functional groups of differentially expressed genes of WT and mutants of *P. inhibens* according to the database of clusters of orthologous groups of proteins (COGs). Up- and down-regulated COGs of each mutant strain with respect to gene expression of the *P. inhibens* WT or the *pgaI* mutant + TDA. Asterisks indicate significantly enriched COG categories. Data for each of the AHL mutants compared to the WT or *pgaI* ^*−*^ + TDA show that the same functional COG categories are up- or down-regulated in a similar number of genes.

Genes that were found differentially expressed in the microarrays were assigned to clusters of orthologous groups (COGs) based on their proposed gene functions (Fig. 2, Table S1), to gain insights into probable functions of QS-regulated gene clusters. Microarray data for each of the AHL mutants compared to the WT or *pgaI* ^*−*^ + TDA show that the same functional COG categories are up- or down-regulated in a similar number of genes (Fig. 2). In all comparisons the largest COG up-regulated in the mutants is cell motility, comprising always 32 genes, including several genes encoding for chemotaxis- and motility-associated proteins. Thus, QS down-regulates motility and chemotaxis genes in the WT. The differential expression of motility genes was verified by qRT-PCR using the *flaF* gene, encoding for the flagellar protein FlaF, as target^26^. This analysis showed that *flaF* gene expression is down-regulated by TDA only in *pgaI* ^*−*^, but not in *pgaR* ^*−*^ (Fig. S2A), indicating that TDA mediates QS only in the presence of the AHL regulator.

**Figure 3:**
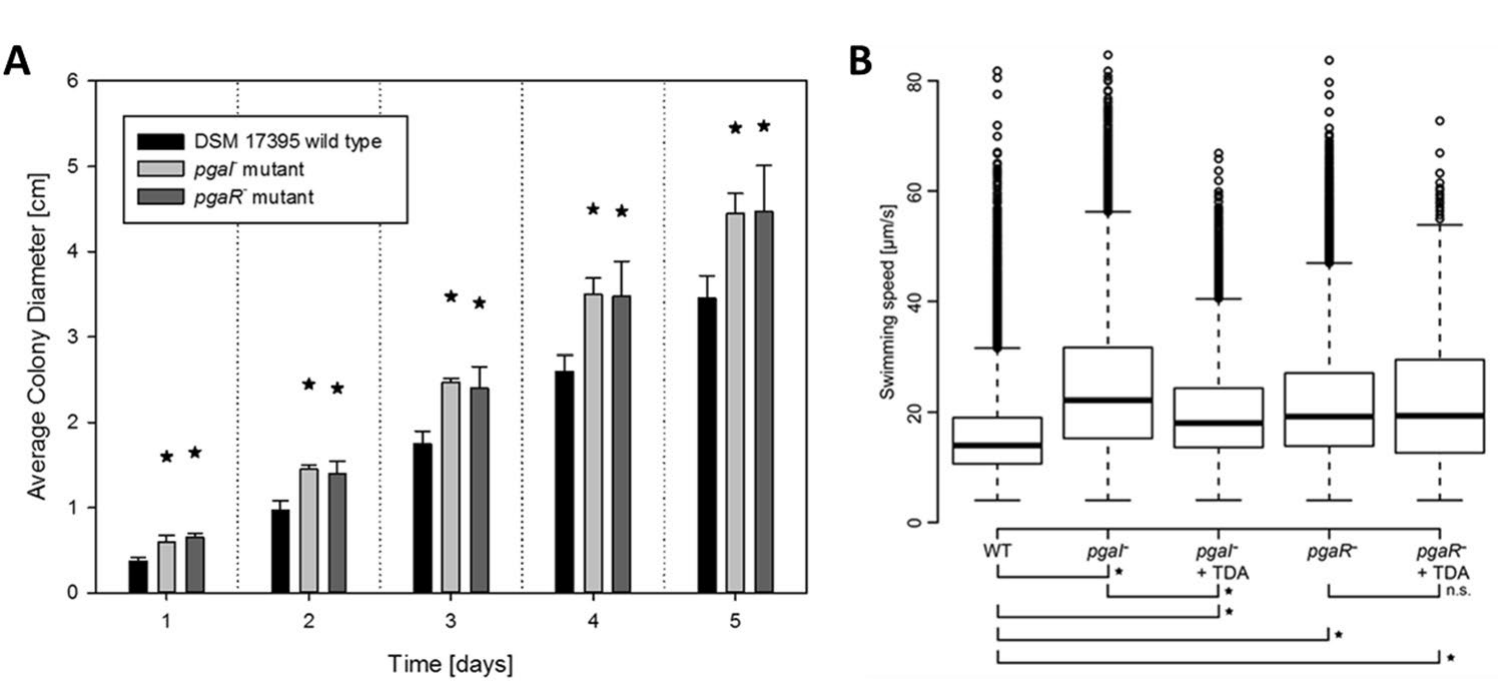
Cell motility tests of the *P. inhibens* WT and the *pgaI* and *pgaR* mutants. (**A**) Motility indicated by colony diameter on soft agar plates over five days. Asterisks indicate significant differences of the mutants compared to the WT on the same day (*p*-value < 0.05). (**B**) Distribution of swimming speeds of individual cells (>1926 trajectories) in liquid MB medium represented as boxplots for *P. inhibens* WT, the *pgaI* ^*−*^ and *pgaR* ^*−*^, and both mutants with 1.5 µM TDA added to the medium (*pgaI* ^*−*^ + TDA and *pgaR* ^*−*^ + TDA). Asterisks indicate significant differences between different strains and conditions, n.s. =not significant (*p* < 0.0001).

Motility assays in soft agar and in liquid medium validated the observation of a down-regulation of the expression of motility genes. Motility experiments in soft agar revealed that *pgaI* ^*−*^ and *pgaR* ^*−*^ were more motile than the WT (Fig. 3A). Furthermore, average swimming speed was measured in liquid medium at the single-cell level (Fig. 3B) to investigate the effect of sub-inhibitory concentrations of exogenous TDA on the motility of the mutants. WT cells swam at 15.9 ± 1.6 µm/s, whereas cells of *pgaI* ^*−*^ and *pgaR* ^*−*^ moved significantly faster, with a speed of 24.2 ± 4.7 and 20.2 ± 2.9 µm/s (*p* < 0.0001), respectively. Cells of *pgaI* ^*−*^ supplemented with exogenous TDA showed a significant reduction in motility (19.3 ± 1.0 µm/s, *p* < 0.0001). In contrast, *pgaR* ^*−*^ cells with exogenous TDA (*pgaR* ^*−*^ + TDA) showed no significant reduction in speed compared to *pgaR* ^*−*^ cells (*p* = 0.44). Both the molecular and the phenotypic data consistently show that motility is down-regulated by QS, which in turn is mediated by AHL and TDA, only in presence of the AHL regulator.

Another COG category down-regulated by AHL- and TDA-mediated QS comprises genes for cell wall/membrane/envelope biogenesis, in which 19 to 25 genes were differentially expressed (Table S1). This includes genes known to play crucial roles in attachment, surface colonization and maintenance of biofilm integrity, e.g., PGA1_c09700, encoding for an N-acetylglucosamine deacetylase and PGA1_c05260, encoding for a sugar transferase, associated with the exopolysaccharide production protein ExoY^27, 28^. Down-regulation of this COG group by QS probably reduces biosynthesis of polar polysaccharides associated with cell attachment in *P. inhibens* ^29^. The strong influence of QS on the genes of this COG group became apparent in an air*-*liquid interface coverslip assay, in which both AHL mutants produced significantly more biofilm than the WT (*p* < 0.001) (Fig. S3). A TDA biosynthesis negative mutant also produced significantly more biofilm than the WT, showing the influence of TDA on biofilm dispersion.

**Figure 4:**
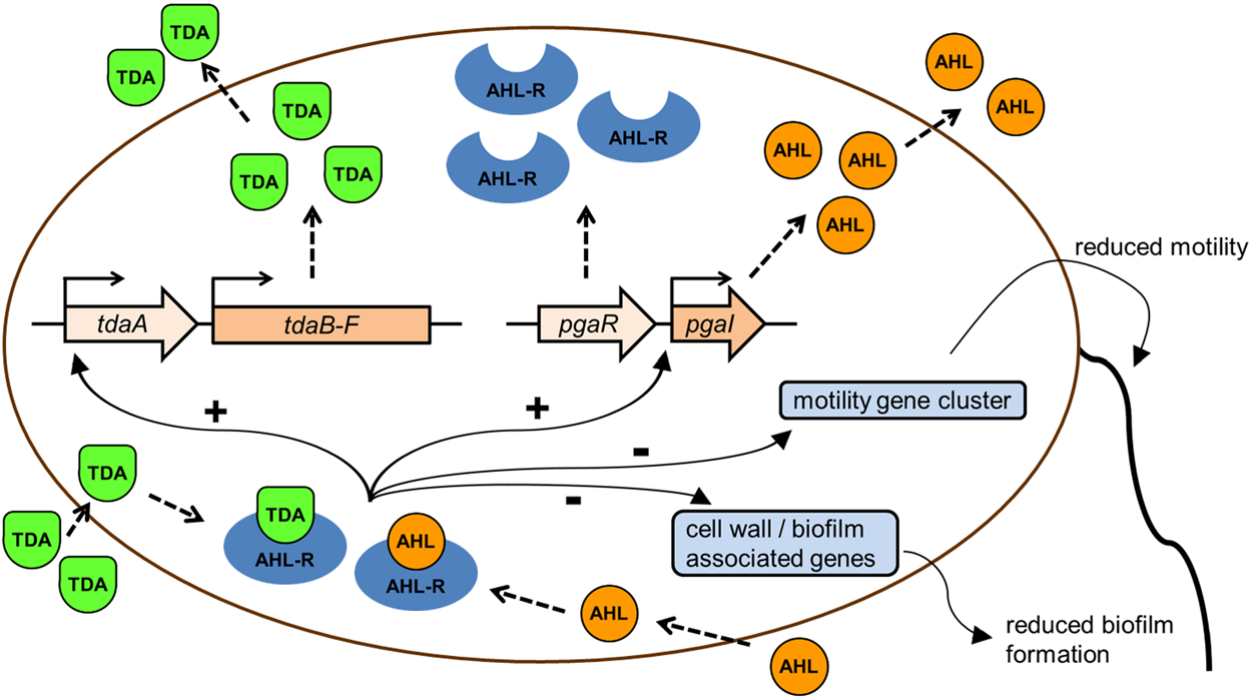
Proposed scheme of quorum sensing signaling in cells of *P. inhibens*. The *pgaR* gene is encoding for the AHL regulator (AHL-R) and *pgaI* for the AHL synthase. AHL as well as TDA molecules interact with the AHL regulator to regulate differential gene expression. QS up-regulates expression of the TDA gene cluster *tdaB-F* and of *tdaA*, an essential transcriptional regulator for the TDA gene cluster, and thus, increases TDA production. Expression of the *pgaI* gene is significantly down-regulated in the *pgaR* mutant compared to WT (Extended Data Table 1), thus upregulated by QS. Therefore, TDA and AHL signaling results in a double positive feedback loop. Genes associated with motility or surface attachment are down-regulated by QS and, consequently, swimming speed and biofilm formation are decreased.

The expression of the TDA biosynthesis gene cluster, *tdaA*-*F*, was down-regulated in the transcriptomes of *pgaI* ^*−*^ and *pgaR* ^*−*^ (Table S1). Using qRT-PCR we verified the microarray result using *tdaA* as target gene, which encodes for the transcriptional regulator of TDA biosynthesis^17^ (Fig. S2B). Addition of TDA significantly increased the *tdaA* expression in *pgaI* ^*−*^ (*p* < 0.01), resulting in similar *tdaA* expression in the WT and *pgaI* ^*−*^ + TDA (*p* = 0.28), but did not change *tdaA* expression in *pgaR* ^*−*^ (*p* = 0.38), validating that TDA needs the AHL regulator for gene regulation. Results from antibiotic bioactivity tests strengthened the transcriptomic results, showing that WT and *pgaI* ^*−*^ + TDA exhibited inhibitory bioactivity against *Pseudoalteromonas tunicata*, with zones of inhibition having diameters of 2.14 ± 0.07 cm and 2.08 ± 0.11 cm, respectively (Table S2). In contrast, *pgaI* ^*−*^, *pgaR* ^*−*^ and *pgaR* ^*−*^ + TDA showed no inhibition (Table S2). These results support the findings from the transcriptomic microarray (Figs 1 and 2; Table S1), the qRT-PCR results (Fig. S2), the motility assays (Fig. 3) and biofilm screenings (Fig. S3) in demonstrating that, in presence of the AHL regulator, AHL and TDA regulate, with the exception of only 15 genes which were differentially expressed in the whole transcriptomes of *pgaI* ^*−*^ + TDA compared to the WT, the same global gene expression (Fig. 4).

## Discussion

As pharmaceutical drugs, antibiotics are typically used in high concentrations to kill pathogenic bacteria, and research has focused primarily on the killing and growth-inhibitory function of antibiotics. Only in recent years antibiotics were shown to interfere with bacterial cell-to-cell communication, yet, how antibiotics affect differential bacterial responses has not been clarified^3–6^. *Pseudomonas aeruginosa* and related bacteria produce quinolones, some of which exhibit antimicrobial activity, however, some quinolones act as quorum-sensing signal molecules, controlling the expression of many virulence genes as a function of cell population density^30^. Antibiotic activity was also found for (7Z)-C14:1-AHL already in the nanomolar range^31^, but no antagonism of the producing bacteria was observed in inhibition assays and this has so far not been studied further. The data presented here provide to the best of our knowledge the first evidence of an antibiotic that regulates QS like an AHL molecule and demonstrate that bacteria can produce antibiotics that substitute their AHL molecules as global gene regulators.

Our finding that a sub-inhibitory concentration of exogenous TDA is able to mediate QS and to restore WT gene expression only in conjunction with the AHL regulator confirmed that TDA interacts specifically with the LuxR-type transcriptional regulator. How exactly TDA and the AHL regulator interact and co-regulate gene expression remains to be determined. The AHL-based QS is obviously located at a high level in the regulation system of *P. inhibens*, controlling the expression of other signaling molecules. For example, five of seven genes associated with c-di-GMP signaling^24^, including three diguanylate cyclases, were negatively regulated by AHL and TDA (Table S1).

In our experimental set-up, results based on WT cells represent the active QS system, as cells were harvested from cultures with high cell numbers, reaching the AHL-threshold for QS. In contrast, the AHL mutants simulate an inactive QS system equivalent to gene expression of single cells or low cell numbers^17^. The AHL- and AHL regulator-deficient strains were highly motile and produced strong biofilms, whereas motility and attachment were reduced in the WT (Fig. 3, Fig. S3). This finding reflects the fact that genes involved in motility, chemotaxis and biofilm formation are more highly expressed in single cells with no activated QS to enhance their ability to find new surfaces, attach and form biofilms (“swim-and-attach”). The importance of motility for the surface attachment was reported for another roseobacter, *Silicibacter* sp. TM1040, by Miller and Belas^32^. This strategy likely provides an advantage for *Phaeobacter* spp. to settle on abiotic and host-associated surfaces, explaining its association with diverse marine eukaryotes, including seahorses, scallop larvae and algae^15, 16, 33^. Down-regulation of motility genes at high population densities by QS was also observed in another alpha-proteobacterium, *Sinorhizobium meliloti*, and it was hypothesized that attached cells do not need high motility and can thereby save energy^34^.

Based on our data, we assume that *P. inhibens* grows on a surface until QS is activated (by the AHL and TDA), whereupon biofilm-associated genes are down-regulated, resulting in considerably reduced attachment. In *Ruegeria* sp. KLH11, another member of the *Roseobacter* group, biofilm dispersion is also induced by AHLs. It has been proposed that this prevents aggregation on crowded surfaces and promotes a balanced colonization of host organisms^35^. Dispersal rates play a key role in determining diversity and function over evolutionary timescales^36^. Therefore, our results point to another explanation, suggesting that *Phaeobacter* is “hitchhiking” on migrating hosts like phytoplankton cells, e.g., the cosmopolitan diatom *Thalassiosira rotula*, on which *Phaeobacter* was previously detected^37^, to access new resources. This can be an important dispersal mechanism, which could help to explain the broad geographic distribution of *P. inhibens* ^15, 36^.

Another reason why QS reduces the attachment of a surface colonizer relates to the “Jekyll-and-Hyde” chemistry of *P. inhibens* suggested by Seyedsayamdost *et al*.^38^, describing a shift from mutualism to pathogenesis through the production of algaecides against its algal host. Killing of the aging host provides rapid access to resources released from the algal cell during its lysis, and QS might induce dispersion and re-association with a new, healthy host. The algaecidal precursors phenylacetyl-CoA and tropolone are also precursors and intermediate products for the biosynthesis of TDA^15^. Based on our finding that TDA mediates QS in *P. inhibens*, we propose that QS can mediate the switch from mutualism to pathogenesis and from attachment to dispersion. That the TDA negative mutant produced more biofilm than the WT (Fig. S3) is probably based on the lacking TDA as QS signaling molecule and demonstrates a role of TDA also in biofilm dispersion.

The capability to produce the antibiotic TDA regulated by QS on the one hand, which mediates QS like AHLs on the other hand, implies that in *P. inhibens* AHL and TDA act simultaneously, probably accelerating QS and adaptation processes, for example those relevant in symbiosis. Furthermore, an organism which produces an antibiotic acting as weapon and as a signaling molecule saves on biosynthesis costs^39^. Since TDA autoinduces its own synthesis in different Roseobacters by cross-feeding^23^, it is likely to act in these and possibly other organisms as a global signal for QS. The probiotic activity of *Phaeobacter* spp. was attributed to TDA production^40^, however, future studies now have to show whether the probiosis is based on the antibiotic activity of TDA only or also its signaling function.

The results presented here, together with prior findings that other antibiotics can interfere with cell signaling^4, 8–10^, indicate that antibiotics at sub-inhibitory concentrations may have considerable impact on the microbiome in natural environments. The widespread use of antibiotics in human medicine, agriculture and aquaculture^2, 7, 41, 42^ might have already changed the microbial world through gene expression regulation by antibiotic signaling. The bulk of the actual impact of antibiotics in the environment has not yet been recognized, because of a lack of understanding and consideration for the role of antibiotics at sub-inhibitory concentrations in QS and, potentially more generally, in inter-microbial signaling.

## Methods

### Marine broth (MB) medium

MB medium was made similar to the commercial Marine Broth 2216 medium (BD Biosciences, #279110), but in comparison shows no precipitate. Chemicals [g/L]: Peptone 5.0, yeast extract 1.0, ferric acid citrate 0.1, MgCl_2_ 6H_2_O 12.6, Na_2_SO_4_ 3.24, NaCl 19.45, CaCl 2H_2_O 2.38, KCl 0.55, NaHCO_3_ 0.16, Na_2_HPO_4_ 2H_2_O 0.01, agar 17.7 (for agar plates only). Chemicals were suspended in purified H_2_O and 10 ml/L of trace element solution ([g/L]: KBr 8.0, SrCl_2_ 6H_2_O 3.4, H_3_BO_3_ 2.2, Na_4_SiO_4_ 5H_2_O 0.7, NaF 0.24, NH_4_NO_3_ 0.16), heated while stirring, boiled for at least one minute and autoclaved afterwards.

### Bacterial strains and growth conditions

Organisms used in this study were *Phaeobacter inhibens* DSM 17395 and three mutant strains of DSM 17395, negative for the AHL synthase gene (*pgaI*, PGA1_c03890, i.e. strain WP38: DSM 17395 *pgaI*::Gm; Gm^r^), the AHL regulator gene (*pgaR*, PGA1_c03880, i.e. strain WP52: DSM 17395 *pgaR*::EZTn*5*; Gm^r^), and a TDA biosynthesis gene (*tdaA*, PGA1_pA00980, i.e. strain WP75: DSM 17395 *tdaA*::EZTn*5*; Gm^r^), respectively^17^. Strains were routinely grown in 500 ml baffled Erlenmeyer flasks at 100 rpm at 28 °C (unless indicated otherwise) with 100 ml MB medium, or on solid agar of half-strength MB medium. If required, media were supplemented with antibiotics to a final concentration of 1.5 µM TDA or 52.3 µM gentamicin sulfate. TDA was obtained from BioViotica Naturstoffe GmbH and dissolved in dimethyl sulfoxide. Each culture was inoculated with ~4.5 × 10^6^ cells of a pre-culture in the late exponential growth phase. To study effects on the gene expression, we analyzed samples from four different time points (early, mid and late exponential, and early stationary growth phase) by preliminary cell enumeration and quantitative reverse transcription-PCR (RTqPCR) experiments. We focussed on samples from the late exponential growth phase, based on most pronounced differences between the different cultures. For microarray and RTqPCR analyses (see below) samples of four biological replicates of each strain were harvested in the late exponential growth phase (~4.5 × 10^9^ cells/ml, Fig. S4), because high AHL and TDA concentrations were observed under these conditions^17^.

### Optical densities and cell enumeration

Growth was measured at an optical density at 600 nm (DU520, Beckman Instruments, Fullerton, USA). For cell enumerations, samples of four biological replicates of *P. inhibens* DSM 17395 wild type (WT), the AHL synthase-deficient mutant (*pgaI* ^*−*^), the AHL regulator-deficient mutant (*pgaR* ^*−*^) and of both mutants grown with exogenous TDA (*pgaI* ^*−*^ + TDA and *pgaR* ^*–*^ + TDA), taken at the late exponential growth, were fixed and aggregates dissolved by sonication, as described in Berger *et al*.^17^. Serial dilutions were used for enumeration via an Accuri C6 Flow Cytometer (Beckton Dickinson). Cells were stained with Sybr Green I (Invitrogen) and detected by blue laser excitation at 488 nm and at wavelength absorbtion of 533+/−15 nm. Growth curves and cell enumerations of the cultures of *P. inhibens* DSM 17395 WT, *pgaI* ^*−*^, *pgaR* ^*−*^, and both mutants grown with TDA added to the medium were compared and indicated that sampling for subsequent gene expression experiments and phenotypic screenings of the different strains are based on the same growth stage and with the same numbers of cells (Fig. S4).

### Antimicrobial activity

Antimicrobial activity against *Pseudoalteromonas tunicata* DSM 14096 was examined by agar diffusion tests as described previously^17^. Inhibition by cultures of *P. inhibens* DSM 17395 and the derived AHL-deficient mutants (*pgaI* ^−^ and *pgaR* ^*−*^), grown with or without exogenous TDA was analyzed in triplicates. Inhibitory zones were measured between the paper disks containing sterile filtered supernatants of bacterial cultures and the bacterial lawn of the target strain.

### RNA isolation

RNA was isolated and purified with the RNAprotect kit (QIAGEN) and the RNeasy kit (QIAGEN), following a modified manufacturer’s protocol^43^. Concentration and purity of RNA was determined by Bioanalyzer (Bioanalyzer 2100, Agilent Technologies) and Nanodrop measurements (NanoDrop ND-2000c, Thermo Scientific). To exclude DNA contamination, each RNA sample was controlled by RTq-PCR (without synthesizing cDNA) using primers for amplification of the 16S rRNA gene (Table S3).

### Microarray design

A customized whole genome microarray was designed using the Agilent earray platform (https://earray.chem.agilent.com/earray/). Three probes for each gene were designed to investigate expression of the whole genome (3960 genes) of *P. inhibens* DSM 17395 and the derived mutants, of 26 internal controls (mainly putative housekeeping genes) and 536 Agilent controls, using the Agilent Technologies Design Wizard for a slide with eight arrays (8 × 15 K). Chosen probe features were length of 60 bp, Antisense, 3′ bias, probes layout randomized and best distribution.

### Microarray experiment

Two-color dual-labeled microarray experiments were performed using a loop design (Fig. S5) with four biological replicates for each strain. For labeling, two µg of total RNA was dyed with Cy3 or Cy5 using the Universal Linkage System^TM^ (ULS) according to the manufacturer’s manual. The degree of labeling must have an average of 1–3.6 Cy-ULS molecules per 100 nt (General/Homebrew Protocol, ULS Labeling Kits, Kreatech). 500 ng labeled RNA of two different strains were fragmented and hybridized to a microarray according to Agilent’s Two-Color microarray protocol (1000-ng total weight). Microarrays were scanned using the Agilent DNA Microarray Scanner. Median foreground and background signals of the Cy3 and Cy5 channel were loaded into the R environment (http://www.cran.r-project.org/) and processed using the LIMMA package^44^ of the BioConductor project (http://www.bioconductor.org/). The *p*-values for differential expression were adjusted for false-discovery rate using the method by Benjamini and Hochberg^45^. Only genes with an adjusted *p*-value ≤ 0.01 and an absolute log2 fold change ≥ 1.0 were considered in the subsequent analyses.

### Microarray data

External Databases S1: Raw and processed microarray data have been deposited in the Gene Expression Omnibus database (http://www.ncbi.nlm.nih.gov/geo/) under accession number GSE79173.

### cDNA synthesis

cDNA for quantitative reverse transcription-PCR (qRT-PCR) experiments was synthesized from isolated RNA by Transcriptor First Strand cDNA Synthesis Kit with procedure B (Roche Diagnostics GmbH, Mannheim). For all samples, negative control reactions were performed by excluding the Transcriptor Reverse Transcriptase and the 30 min heating step at 55 °C. Additionally, water as a second negative control and a mix of all RNA samples as a positive control, were used as templates.

### Quantitative reverse transcription-PCR

Verification of differentially expressed genes detected in the microarray experiments was performed by a qRT-PCR calibrator-normalized relative quantification with efficiency correction (E_T_^CpT (C)–CpT (S)^ × E_R_^CpR (S)−CpR (C)^) in the LightCycler^®^ 480II system (Roche Diagnostics GmbH), version 1.5. Relative quantification was performed as described in the LightCycler^®^ 480 Instrument Real-time PCR Protocol for 96-well plates (Operators Manual) by using probes of the Universal ProbeLibrary and specific primer pairs, designed via the Universal ProbeLibrary Tool by uploading the complete target gene sequence (Roche Diagnostics). Standard curves for target and reference genes were created using dilution series with five 10-fold dilutions of a cDNA mix, prepared by cDNA synthesis with a mix of all RNA isolations as a template. A two-fold dilution of this cDNA mix was used as a calibrator. qPCRs for all samples were run with primers for target genes and with primers for the 16S rRNA gene sequence as a reference (Table S3). All values were normalized relative to the calibrator expression performed with the same primers. Water and negative controls originated from the cDNA synthesis protocol were used as negative controls. All results were gained by using three or four biological replicates and shown as calibrator-normalized ratio of target/reference concentrations with efficiency control.

### Air-Liquid Interface Glass Coverslip Assay

Three biological replicates of each strain were inoculated in 20 ml MB medium with an OD^600^ of 0.05 in 50 ml falcon tubes containing glass slides, shaken gently at 10 rpm in a 45° angle. An OD^600^ of 0.05 corresponds for all strains to a cell number of ~1 × 10^8^ cells/ml (cell enumeration was performed by flow cytometry as described above). After two days, glass slides were washed four times with sterile water, biofilms were stained with crystal violet and dissolved in 23 ml modified biofilm dissolving solution (sodium dodecyl sulphate solution dissolved to a final concentration of 10% with 80% ethanol in H_2_O), and optical density was measured at a wavelength of 590 nm and subtracted by values of controls without bacteria^46, 47^.

### Motility test

Swimming motility was tested on soft-agar plates (3 g l^−1^ agar) with 10% MB medium at 28 °C, based on the method of Miller and Belas^32^. Chemicals were dissolved in sterile-filtered natural seawater, sampled in the southern North Sea, Germany (taken at position 54°0′0,0″N 8°9′21,6″E, 4.5 m depth, on 30^th^ September 2011). Motility measurements were done on agar plates, inoculated with cells of the outer ring of previous soft-agar plates with the same medium and culture conditions. Swimming was measured for six days.

Swimming speed was also measured in microfluidic channels using phase-contrast microscopy (Nikon Ti-E microscope; 20×, 0.45 NA objective)^48^. For microfluidic experiments, strains were grown in 15 ml tubes at 30 °C with 3 ml MB medium. Strains were collected at late exponential phase and diluted 5~10 fold in fresh MB media (or fresh MB media with TDA) before single-cell level bacterial tracking. Bacteria were imaged at mid depth in a 120 µm deep polydimethylsiloxane (PDMS) chamber at 17~30 frames/s using Cooke PCO 1600 CCD camera (7.4 µm/pixel). All image analyses and cell trackings were performed in Matlab (The Mathworks) using in-house, automated software to reconstruct cell trajectories and calculate mean speeds^48^.

## Electronic supplementary material

Supplementary Information

Supplementary Table S1
