# Supplementary material for: Dual function of tropodithietic acid as antibiotic and signaling molecule in global gene regulation of the probiotic bacterium Phaeobacter inhibens
Source: Sci Rep. 2017 Apr 7;7:730. doi: 10.1038/s41598-017-00784-7 (PMC5429656; doi:10.1038/s41598-017-00784-7)
Supplement: Supplementary file 1 — Supplementary Information [file 41598_2017_784_MOESM1_ESM.pdf]

Supplementary Information

Dual function of tropodithietic acid as antibiotic and signaling molecule in global gene regulation of the probiotic bacterium *Phaeobacter inhibens*

Paul G. Beyersmann[1], Jürgen Tomasch[2], Kwangmin Son[3], Roman Stocker[3,4], Markus Göker[5], Irene Wagner-Döbler[2], Meinhard Simon[1], and Thorsten Brinkhoff[1,*]

[1] Institute for Chemistry and Biology of the Marine Environment (ICBM), University of Oldenburg, 26129 Oldenburg, Germany.

[2] Helmholtz Centre for Infection Research, 38124 Braunschweig, Germany.

[3] Department of Civil and Environmental Engineering, Massachusetts Institute of Technology, Cambridge, MA 02139-4307, USA.

[4] Institute for Environmental Engineering, Department of Civil, Environmental and Geomatic Engineering, ETH Zurich, 8093 Zurich, Switzerland

[5] Leibniz-Institute DSMZ – German Collection of Microorganisms and Cell Cultures GmbH, 38124 Braunschweig, Germany.

[*]Correspondence and requests for materials should be addressed to T. B. (email: t.brinkhoff@icbm.de)

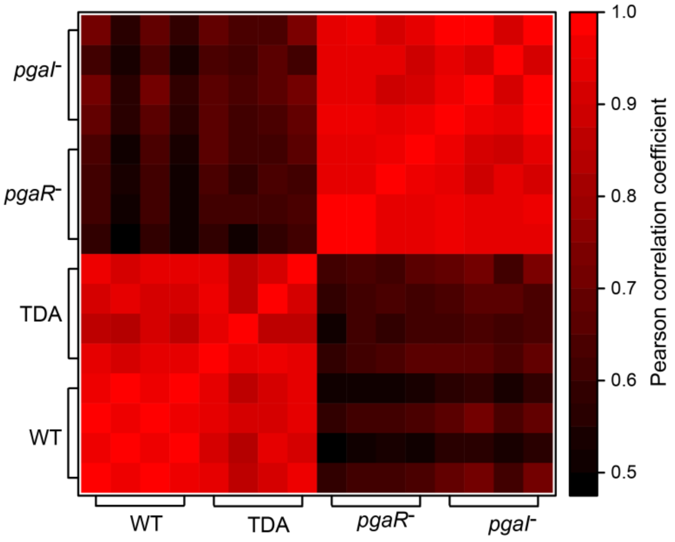

Fig. S1. Heat map showing the correlation of the transcriptomes of *P. inhibens* DSM 17395 WT, the QS negative mutants (*pgaI*$^-$ and *pgaR*$^-$) and the *pgaI* mutant with exogenous TDA. Coloration is based on the pairwise Pearson correlation coefficients of the microarray data. Red color indicates high correlation, black color low correlation.

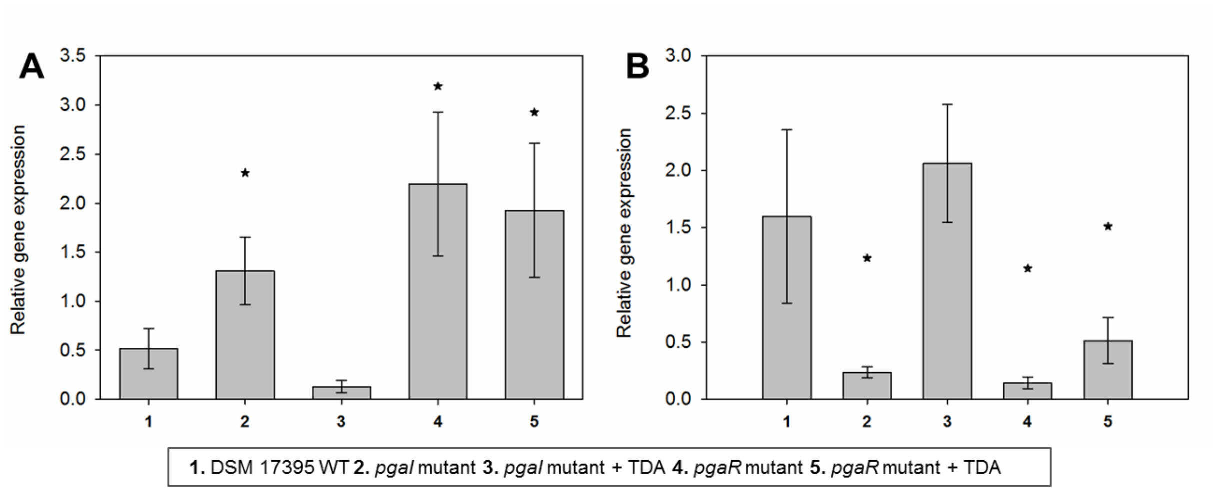

Fig. S2. Validation of microarray gene expression results obtained for *P. inhibens* DSM 17395 by qRT-PCR, determining expression levels of (A) *flaF* (PGA1_c35860), encoding for the flagellar protein FlaF[26], and (B) *tdaA* (PGA1_262p00980), encoding for the transcriptional regulator of TDA biosynthesis[17]. Relative quantification was based on expression of the 16S rRNA gene as reference. Expression levels were determined for *P. inhibens* DSM 17395 WT, the QS negative mutants *pgaI* and *pgaR*, and for both mutants grown with exogenous TDA. Asterisks indicate significant different relative gene expression compared to the *P. inhibens* DSM 17395 WT (p-value < 0.05).

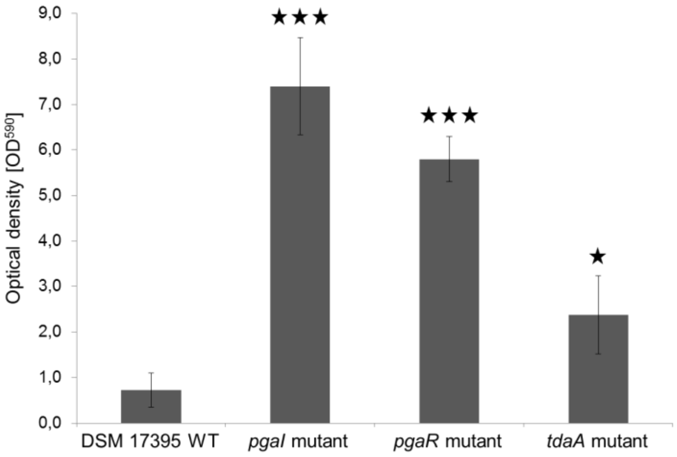

Fig. S3. Cell attachment of the *P. inhibens* WT, the *pgaI* mutant, the *pgaR* mutant and the *tdaA* mutant on glass coverslips in the air-liquid interface. Biofilms were stained with crystal violet, dissolved by MBDS and optical density was measured at a wavelength of 590 nm. Asterisks show significant differences between *P. inhibens* DSM 17395 WT compared to the *pgaI* mutant, *pgaR* mutant and *tdaA* mutant (three asterisks: p-value < 0.001, one asterisk: p-value < 0.05).

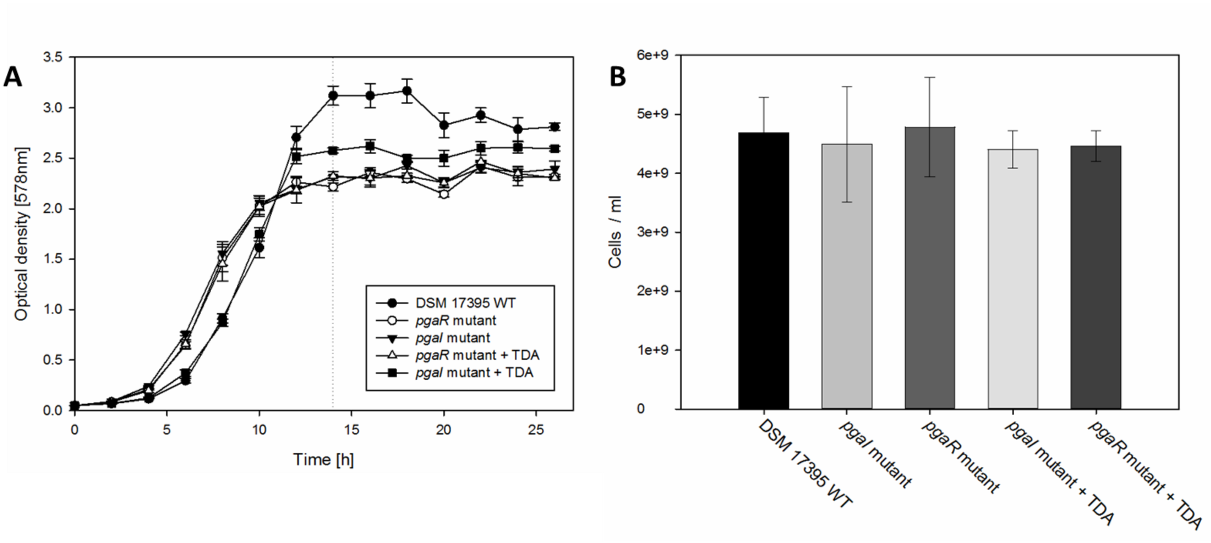

Fig. S4. (A) Growth curves and (B) cell enumeration of *P. inhibens* DSM 17395 WT, *pgaI*$^{-}$, *pgaR*$^{-}$, and both mutants grown with TDA added to the medium [1.5 µM]. (A) Vertical dotted line indicates the sampling for cell counting and RNA extraction for subsequent qRT-PCR and microarray experiments. Higher optical densities of the WT and *pgaI*$^{-}$ + TDA in late exponential growth are due to a stronger brownish pigmentation, but not due to higher cell numbers[17]. Addition of sub-inhibitory concentrations of TDA restored the dark pigmentation of the WT in the *pgaI*$^{-}$, but not in *pgaR*$^{-}$. Thus, the *pgaR*$^{-}$ + TDA was not included in the whole transcriptome analyses, but specific gene expression was examined by RT-qPCR.

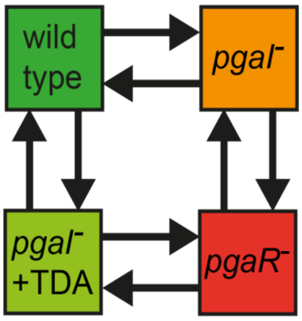

Fig. S5. Microarray loop design for comparison of differential gene expression of 16 biological samples used in eight two-color dual-labeled microarrays (Agilent GE 8x15K).

Additional Data Table S1 (separate file):

Table S1. Whole transcriptome comparisons based on the microarray data. The file offers one sheet “All microarray results”, which allows an overview of the transcriptome data and sheets for each transcriptome comparison, i.e. *pgaI*$^{-}$ vs WT, *pgaR*$^{-}$ vs WT, *pgaI*$^{-}$ + TDA vs WT, *pgaI*$^{-}$ vs *pgaI*$^{-}$ + TDA, *pgaR*$^{-}$ vs *pgaI*$^{-}$ + TDA and *pgaI*$^{-}$ vs *pgaR*$^{-}$. One column shows the transcriptomic fold change value, which represents the fold change in the first mentioned transcriptome compared to the second transcriptome, and another column shows the significance of possible differences (*p*-value) between the two transcriptomes for each microarray. The table includes a column for functional groups of differentially expressed genes according to the database of clusters of orthologous groups of proteins (COGs).

Table S2. Antimicrobial activity of *P. inhibens* DSM 17395 WT and the derived AHL-deficient mutants (*pgaI*$^{-}$ and *pgaR*$^{-}$), grown with or without exogenous TDA (*pgaI*$^{-}$ + TDA and *pgaR*$^{-}$ + TDA) against *Pseudoalteromonas tunicata* DSM 14096 cultures. Numbers represent the zone of inhibition measured in cm and the minus sign indicates that no inhibition was observed. Antimicrobial tests were done in nine biological replicates.

biological replicate #	zones of inhibition in diameters [cm]				
	WT	*pgaI*$^{-}$	*pgaI*$^{-}$ + TDA	*pgaR*$^{-}$	*pgaR*$^{-}$ + TDA
1	2.22	-	2.16	-	-
2	2.10	-	2.16	-	-
3	2.16	-	2.10	-	-
4	2.10	-	2.22	-	-
5	2.16	-	1.81	-	-
6	2.28	-	2.10	-	-
7	2.04	-	1.99	-	-
8	2.04	-	2.10	-	-
9	2.16	-	2.04	-	-
**mean**	**2.14**	**-**	**2.08**	**-**	**-**
**standard derivation**	**0.07**	**-**	**0.11**	**-**	**-**

Table S3. Primer and hydrolysis probes used in this study for qRT-PCR. The probes were obtained from the Universal Probe Library (UPL) of the Roche Diagnostics GmbH.

Gene	gene ID	locus tag	UPL probe #	forward primer 5'-3'	reverse primer 5'-3'
16S rRNA	2510178581	PGA1_c00110	104	CGCAACCCACATCCTTAGTT	TATCACGGGCAGTTTCCCTA
tdaA	2510182280	PGA1_262p00980	47	CGGATCTGGAAGTCGCTTT	CGTTGCGAATATCGTCCA
flaF	2510182119	PGA1_c35860	108	ACTCCCGGACGAATTGAAA	TGGTGTGTTGATGGGTGAAT

Additional Databases S1 (separate file):

Raw and processed microarray data have been deposited in the Gene Expression Omnibus database (http://www.ncbi.nlm.nih.gov/geo/) under accession number GSE79173.
